# Optogenetics-enabled assessment of viral gene and cell therapy for restoration of cardiac excitability

**DOI:** 10.1038/srep17350

**Published:** 2015-12-01

**Authors:** Christina M. Ambrosi, Patrick M. Boyle, Kay Chen, Natalia A. Trayanova, Emilia Entcheva

**Affiliations:** 1 Department of Biomedical Engineering, Stony Brook University, Stony Brook, NY; 2 Institute for Computational Medicine and Department of Biomedical Engineering, Johns Hopkins University, Baltimore, MD.

## Abstract

Multiple cardiac pathologies are accompanied by loss of tissue excitability, which leads to a range of heart rhythm disorders (arrhythmias). In addition to electronic device therapy (i.e. implantable pacemakers and cardioverter/defibrillators), biological approaches have recently been explored to restore pacemaking ability and to correct conduction slowing in the heart by delivering excitatory ion channels or ion channel agonists. Using optogenetics as a tool to selectively interrogate only cells transduced to produce an exogenous excitatory ion current, we experimentally and computationally quantify the efficiency of such biological approaches in rescuing cardiac excitability as a function of the mode of application (viral gene delivery or cell delivery) and the geometry of the transduced region (focal or spatially-distributed). We demonstrate that for each configuration (delivery mode and spatial pattern), the optical energy needed to excite can be used to predict therapeutic efficiency of excitability restoration. Taken directly, these results can help guide optogenetic interventions for light-based control of cardiac excitation. More generally, our findings can help optimize gene therapy for restoration of cardiac excitability.

Classic gene and cell therapies to restore or improve cardiac tissue excitability *in vivo* involve the administration of either viral vectors or cells carrying the gene of interest through direct myocardial injection or through coronary or systemic perfusion^1^. The injection delivery method has been shown to result in a clustering of transgene expressing cells^2^,^3^,^4^, while the systemic approach yields a more diffuse pattern of expression^5^,^6^. No systematic study exists comparing the mode of delivery—viral gene (GD) or cell (CD)—and the administration-specific geometric constraints, specifically the spatial patterns of expression (clustered or diffuse), as factors influencing the functional outcomes of these therapies, many of which are in clinical trials^7^,^8^,^9^. Although the spatial distribution of transduced cells can be revealed using fluorescent reporter proteins (e.g., GFP) or other tags^10^, *it is currently not possible to directly, selectively, and exclusively engage only the transgene-expressing cells to assess their exact contribution to the functional tissue response (specifically, the resulting modulation of bioelectric properties)*. Quantification of the functional engagement of transduced cells is a prerequisite for evaluating the efficiency of and optimizing the delivery parameters for gene and cell therapies.

In tissue that has been optogenetically modified to express an excitatory opsin, such as channelrhodopsin-2 (ChR2)^11^, it is possible to functionally engage only the transduced cells using light. We and others have demonstrated that genetic modification of heart tissue to express light-sensitive proteins (opsins) enables control of cardiac activity at low energies with high spatial and temporal resolution^6^,^12^,^13^,^14^,^15^,^16^,^17^,^18^,^19^,^20^,^21^,^22^,^23^,^24^. Energy requirements for optogenetic stimulation depend on both electrophysiological properties of light-sensitive cells and the spatial distribution of ChR2-expressing units in illuminated tissue^25^, but this complex relationship has not been fully characterized. For well-coupled cardiac tissue that has been optogenetically transformed, the ability to *functionally engage* only transduced cells by light allows for quantitative assessment of the optimality of the spatial distribution of the transduced cells and their functional contribution, hence the efficiency of the “therapy”. We hypothesize that ChR2 expression can be used to abstract transduction with excitatory membrane proteins for the purpose of restoring excitability; that is, the threshold irradiance to achieve global excitation in optogenetically-transformed tissue can serve as a quantitative indicator of therapeutic efficiency of gene or cell therapy across delivery modes and geometric patterns. As such, if a particular optogenetic configuration, namely: the combination of delivery mode and spatial pattern of transduced cells, results in a lower optical excitation threshold, then restoration of excitability via gene or cell delivery with an analogous configuration could be deemed more efficient. Notably, the optogenetic approach appears unique in addressing this particular research question because no alternative experimental methodology exists that is capable of characterizing the functional consequences of each different pattern of cell arrangement.

We present here an optogenetics-based strategy to quantify the efficiency of gene and cell therapy, linking delivery mode (GD or CD) and spatial arrangements of a transgene (abstracted by the excitatory opsin ChR2) to functional (bioelectric) tissue response, represented by an increase in global excitability. Using *in vitro* experiments and *in silico* analysis of a wider range of conditions, we derive a general framework of principles governing the relationship between delivery mode, spatial distribution of ChR2-expressing cells, and optical energy required to elicit excitation. We validate the proposed framework by demonstrating that the performance of different transgene configurations (via addition of exogenous sodium channels to tissue with impaired excitability) is correctly predicted by our optogenetics-based analysis. These findings provide insights for the rational design of future biological interventions, including *in vivo* optogenetic manipulations, as well as more general non-optogenetic therapies for the restoration of cardiac excitability.

## Results

### Gene and cell delivery with spatially distinct transgene distributions: *in vitro* and in silico

We used a simple *in vitro* model of cardiac syncytium (neonatal rat ventricular cardiomyocytes), in which cells were transduced by either viral gene delivery (GD) or cell delivery (CD) to express an opsin, ChR2, in three spatially distinct patterns. Transgene distributions were designed to mimic the results of physiologically-realistic delivery modalities: 1) a consolidated, localized island of expression resulting from direct intra-myocardial injection^2^,^3^,^26^; 2) a spatially-disperse, low-density distribution produced by low-efficiency systemic or pericardial sac delivery^27^; and 3) a spatially-uniform, high-density expression pattern resulting from high-efficiency systemic transduction^5^,^6^ (often desirable as *in vivo* transduction).

**Figure 1:**
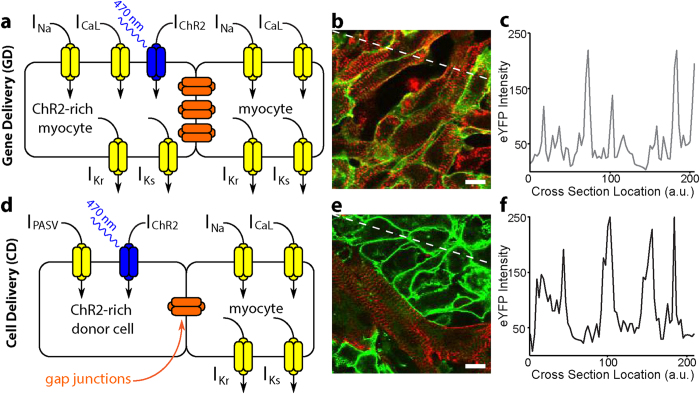
Inscription of light sensitivity by viral gene or cell delivery. (**a**) Schematic for gene delivery of (GD) of ChR2. In cardiomyocytes (CMs) where ChR2 transduction was successful (left), channels that conduct light-sensitive current (I_ChR2_; blue) are trafficked to the sarcolemma; these cells are connected via gap junctions (orange) to normal, non-transduced heart cells (right), as in unmodified tissue. (**b**) Superimposed immunofluorescence images of cardiac cell monolayers with light-sensitivity inscribed by GD showing co-localization of markers for CMs (α-actinin, red) and ChR2 (eYFP, green). (**c**) EYFP intensity profile of ChR2-expressing CMs along the cross section indicated by the dotted white line in (**b**). (**d**) Schematic for cell delivery (CD) of ChR2. ChR2-rich donor cells (left) co-cultured with normal CMs (right) form a light-sensitive heterocellular monolayer. Membrane behaviour in donor cells is considered passive and represented by a leakage current (I_PASV_) with properties based on experimental measurements from HEK cells. (**e**) Superimposed immunofluorescence images of monolayers with light-sensitivity inscribed by CD showing distinct, adjacent CMs (red) and ChR2-expressing donor cell (green) regions. (**f**) EYFP intensity profile of ChR2-expressing donor cells along the cross section indicated by the dotted white line in (**e**). Generic cardiac ion channels (yellow) are shown in CM schematic. Scale bars in (**b**,**d**) are 10 μm.

As illustrated in Fig. 1a,b, in the case of GD, cardiomyocytes were directly infected with a custom-made adenovirus containing the transgene for ChR2-eYFP. Opsin-expressing cardiomyocytes then formed gap junctions with other cardiomyocytes (both opsin-expressing and unmodified)^18^. In the case of CD (Fig. 1d,e), optical sensitivity was achieved by incorporating ChR2-rich somatic (HEK) cells within the syncytium. The donor (HEK) cells, which were light-sensitive and conductive, but non-excitable, formed gap junctions with native, unmodified cardiomyocytes^14^. Regardless of delivery method (GD or CD), ChR2 was expressed as a membrane-specific ion channel (Fig. 1b,e, green). eYFP intensity profiles showed that the expression of the reporter (as a surrogate for ChR2 expression) was similar between transduced myocytes (GD) and ChR2-expressing donor cells (CD) in syncytial distributions (Fig. 1c,f).

**Figure 2:**
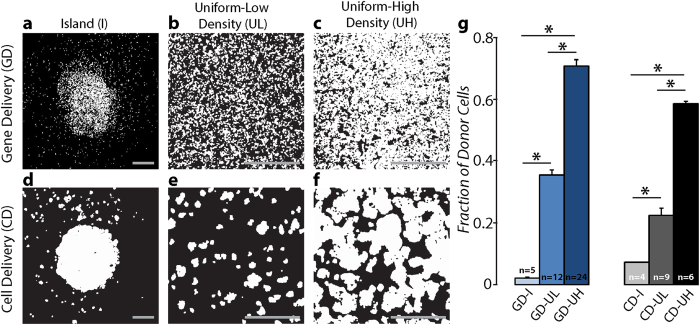
Patterned light-sensitive cardiac syncytia *in vitro*. (**a–c**) Binarized panoramic images (based on eYFP fluorescence) showing the island (I), uniform low (UL), and uniform high (UH) spatial distributions of light-sensitive (white) and non-transduced (black) myocytes in monolayers with light sensitivity inscribed by gene delivery (GD). (**d–f**) Same as (**a–c**) but showing distributions of ChR2-rich donor cells (white) and myocytes (black) in monolayers with light sensitivity inscribed by cell delivery (CD). (**g**) Fraction of monolayer (by area) consisting of light-sensitive cells for the six configurations shown in (**a–f**). Data are presented as mean ± standard error of the mean. Scale bars are 1 mm.

Experimentally, gene patterning techniques and gene titration (see Ambrosi *et al.*^18^ and the Materials and Methods section) were used for both delivery modalities (GD and CD) to obtain three principal transgene configurations: a consolidated island (I; Fig. 2a,d); a spatially uniform, low-density distribution (UL; Fig. 2b,e); and a spatially uniform, high-density distribution (UH; Fig. 2c,f). These distributions represent a range of opsin densities (D), as quantified in two dimensions (Fig. S1 and **Movie S1**), with ratios ranging from 0.02 ± 0.002 (GD-I) to 0.71 ± 0.02 (GD-UL) based on binarized versions of the imaged eYFP intensities (Fig. 2g). In addition to the transgene intensity profiles of the cells (Fig. 1c,f), the fraction of transduced cells (from the binarized images) was similar (not significantly different) between GD and CD configurations (e.g. 0.36 ± 0.02, GD-UL; 0.23 ± 0.02, CD-UL), allowing for direct comparisons of optical excitability between delivery modes.

**Figure 3:**
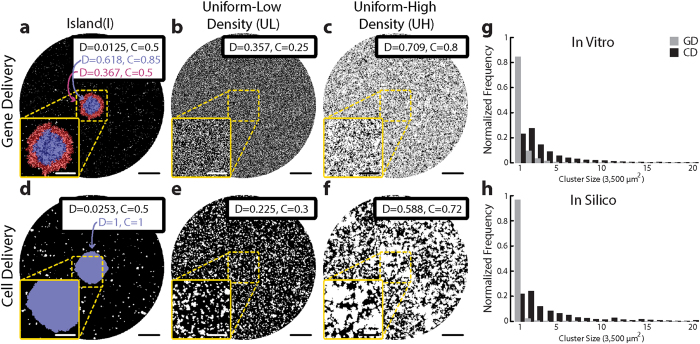
Patterned light-sensitive cardiac syncytia in silico. (**a–f**) Computational models of light-sensitive cell monolayers with light sensitivity inscribed by gene delivery (GD; **a–c**) or cell delivery (CD; **d–f**). Heterogeneous spatial distributions of ChR2-expressing cells (transduced myocytes in (**a–c**), donor cells in (**d–f**)) were generated using a previously-described stochastic algorithm^25^; for each distribution type (I, UL, UH) and delivery mode (CD or GD), parameter values for density and clustering (D and C; shown in black boxes) were chosen to ensure a good match between *in silico* and *in vitro* monolayers (compare (**a–f**) to Fig. 2a–f) For GD-I (**a**), the algorithm was invoked with distinct parameter combinations in 3 distinct regions: the central target (blue shaded; 1.6% of total monolayer area), an outer ring (red shaded; 1.3% of monolayer area), and the surrounding area. For CD-I (**d**), model generation involved two invocations of the distribution algorithm: first, to produce a large central cluster (blue; 4.5% of monolayer are); second, to deliver light-sensitive cell clusters to the peripheral region. (**g**) Normalized histogram showing occurrence rates of light-sensitive cell cluster sizes for *in vitro* monolayers of the UL spatial distribution; histograms for GD (Fig. 2b) and CD (Fig. 2e) modes are compared. (**h**) Same as (**g**), but for *in silico* cell monolayer models. Main panel scale bars in (**a–f**) are 2 mm; inset panel scale bars are 1 mm.

Image-based templating and a stochastic algorithm^23^,^28^ with additional regularization steps were used to derive parametric computational representations of the experimentally-designed transgene distributions; spatial patterns representing a much wider range of conditions than feasible to characterize experimentally were also generated and analysed. The parametric *in silico* representations used opsin density (D) and clustering (C) parameters (see Materials and Methods section), tuned to faithfully capture the experimental *in vitro* transgene distributions, the latter obtained using high-resolution panoramic confocal images (compare Fig. 2a–f and Fig. 3a–f). The density (D) parameter determined the proportion of opsin-expressing units by volume, while clustering (C) quantified the degree of aggregation of light-sensitive regions.

Histograms of transgene cluster sizes for the GD-UL and CD-UL *in vitro* configurations (Fig. 3g) reflected the tendency of light-sensitive cells in CD mode to aggregate into larger clusters (black bars), whereas opsin-expressing cells in GD tended to be more diffuse, forming smaller clusters (grey bars). The corresponding *in silico* distributions (Fig. 3h) closely matched these features, suggesting that the generated models were suitable for simulating the experimentally observed behaviour. The functional aspects (membrane kinetics representation) of GD and CD optogenetic transformation of cardiac syncytia by ChR2 were captured *in silico* using previously described methods^16^,^23^,^25^.

### Optogenetic modification and actuation does not interfere with syncytial properties

**Figure 4:**
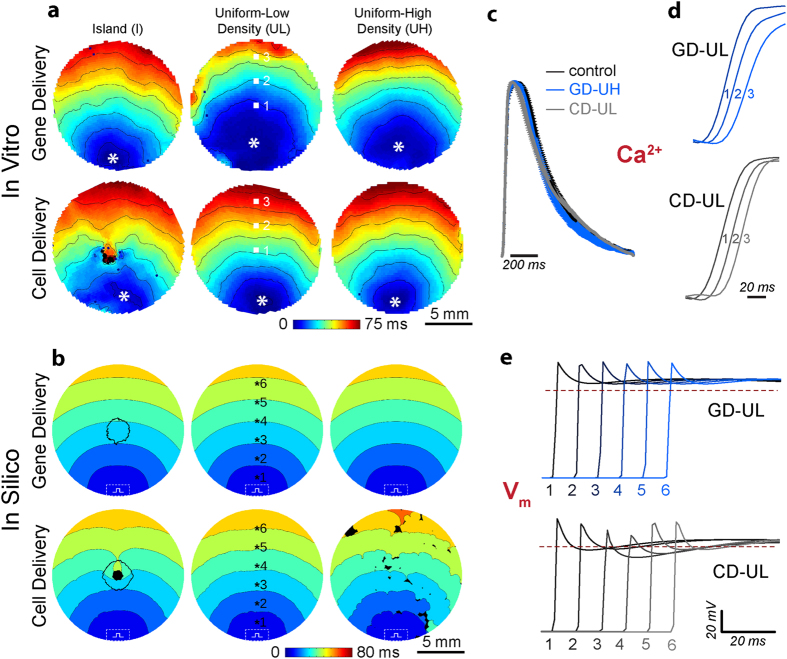
Conduction properties of light-sensitive cardiac syncytia. (**a,b**) Activation maps during electrical stimulation (1 Hz) of *in vitro* (**a**) and *in silico* (**b**) light-sensitive cell monolayers with distribution types (I, UL, UH) and delivery mode (GD, CD) corresponding to the same lettered panels in Figs 2 and 3. In all cases, time zero corresponds to the start of the electrical stimulus; black coloured locations did not activate. For *in vitro* cases, the asterisk (*) marks the location of the bipolar pacing electrode and the spacing between isochrones lines is 10 ms. For *in silico* cases, the dashed rectangle indicates the area where transmembrane current stimulus was applied. (**c**) *In vitro* calcium transients at 1 Hz electrical pacing, plotted overlaid as group mean ± SEM, for control CMs, ChR2-expressing CMs (GD-UH), and co-cultures of CMs and ChR2-expressing HEK cells (CD-UL) (n = 5 per group); no significant differences. (**d**) Select calcium transients from the pixel locations 1–3 indicated in ((**a**), GD-UL and CD-UL) showing *in vitro* wavefront propagation across the monolayer. (**e**) Select voltage traces (analogous to those in (**c**)) from the pixel locations 1–6 indicated in (**b**, GD-UL and CD-UL) showing *in silico* wavefront propagation and upstroke morphology.

Using optical mapping, combined with electrical or optical stimulation, we first experimentally tracked the functional bioelectric responses of the cardiac monolayers: 1) to confirm that the optogenetic transduction did not interfere with function beyond light-induced excitation, and thus ascertain that the experimental system is suitable for assessing the effects of a transgene’s spatial distribution; and 2) to validate the functional predictions of the corresponding *in silico* models (with matching D and C parameters). Upon electrical stimulation (1 Hz, 5 ms pulses), for each of the six transgene spatial distributions, activation patterns for the *in vitro* and *in silico* cases showed similarities at both the macroscopic and microscopic scales (Fig. 4). For GD, the presence of ChR2 within excitable cardiomyocytes did not disrupt conduction (Fig. 4a,b, top row); in contrast, as expected, the presence of ChR2-expressing, non-excitable somatic cells for CD resulted in heterogeneous conduction patterns (Fig. 4a,b, bottom row), particularly in the case of clustered donor cells in CD-I (Fig. 4a,b, bottom left). Overall, there were no statistically significant differences between the *in vitro* and *in silico* macroscopic conduction velocities (CVs at 30 ± 0.5 °C, 18.4 ± 0.7 cm/sec vs 22.7 ± 0.3 cm/sec, respectively) for different delivery modes and/or transgene patterns (Fig. S2).

Detailed analysis of experimentally obtained calcium transients revealed no significant morphological differences between control (i.e., non-transduced) CMs, ChR2-expressing CMs (GD-UH) and CMs coupled to ChR2-expressing donor cells (CD-UL) (Fig. 4c). Transients from spatially distinct pixel locations 1–3 (as indicated on the CD-UL and GD-UL maps in Fig. 4a) showed the expected sequential activation, reinforcing that optogenetic modulation of the cardiac syncytium did not interfere with the baseline bioelectric response. *In silico* analysis of action potential propagation sequences (membrane voltage traces from locations 1–6 in GD-UL and CD-UL, Fig. 4d) revealed subtleties that were not apparent in calcium transients mapped *in vitro*. Specifically, for CD cases, there were distinct amplitude differences in regions where the electrotonic load from coupled ChR2-rich (but intrinsically non-excitable) donor cells was large (Fig. 4e).

**Figure 5:**
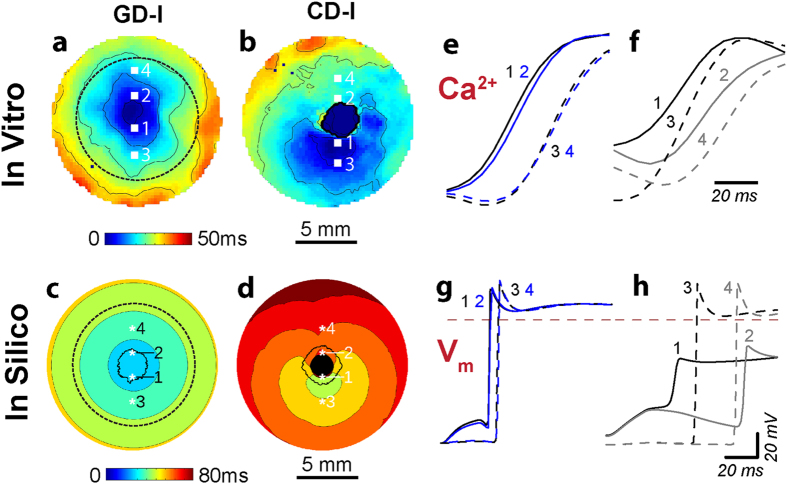
Response to optical stimulation in light-sensitive cardiac syncytia. (**a,b**) Activation maps resulting from optical stimulation (1 Hz) of *in vitro* light-sensitive cell monolayers in the island configuration. Optical stimulus strength was at most 0.07 mW/mm^2^ greater than the threshold irradiance required to elicit a propagating response (E_e,thr_). Time zero corresponds to the beginning of a 20 ms-long pulse of blue light (wavelength λ = 470 nm) applied to the 1 cm-diameter region indicated by the dashed black line in (a); spacing between isochrones is 10 ms. (**c,d**) Same as (**a,b**) but for *in silico* cell monolayers. Simulated optical stimuli were at most 0.0005 mW/mm^2^ greater than E_e,thr_. Here time zero corresponds to the end of each 20 ms-long illumination pulse instead of the beginning; spacing between isochrones is 10 ms. Black-coloured locations did not activate. (**e,f**) Select *in vitro* calcium transients from the pixel locations 1–4 indicated in (**a,b**) on opposite sides of the island of ChR2-expressing donor cells (CM in GD and HEK in CD) showing the wavefront activation sequence. (**g,h**) Select *in silico* voltage traces (analogous to those in (**e,f**)) from locations 1–4.

Optical excitation (1 Hz, 20 ms pulses) of light-sensitive cardiac monolayers resulted in similar patterns of homogenous global conduction with earliest activations occurring near the centre of the 1 cm diameter illuminated area (dotted black line in Fig. 5a,c). As with electrical activation (Fig. 4a,d), the CD-I configuration resulted in heterogeneous conduction patterns with the earliest excitation occurring consistently at the interface between ChR2-expressing, non-excitable donor cells and surrounding cardiomyocytes (Fig. 5b). This conduction pattern was corroborated *in silico*, where the coupling of ChR2-expressing donor cells and cardiomyocytes was an important determinant of optical excitability (Fig. 5d). The homogeneity of light-induced activation patterns for GD compared to CD cases was equally evident in propagation sequences of *in vitro* calcium transients (Fig. 5e,f) and *in silico* voltage traces (Fig. 5g,h). As observed previously, the electrotonic effect from non-excitable donor cells was clearly discernible in CD cases, especially in voltage signals from regions near the border of the central island of ChR2-expressing cells (i.e., traces 1 and 2 in Fig. 5h). Activation patterns and propagation sequences for the island configuration (Fig. 5) were conceptually consistent with UL and UH distributions for GD and CD, in that excitations originated from the centres of regions with densely-expressed ChR2 for GD and from the edges of ChR2-rich donor cell clusters for CD.

### Optogenetics-enabled assessment of GD and CD approaches to restore cardiac excitability

**Figure 6:**
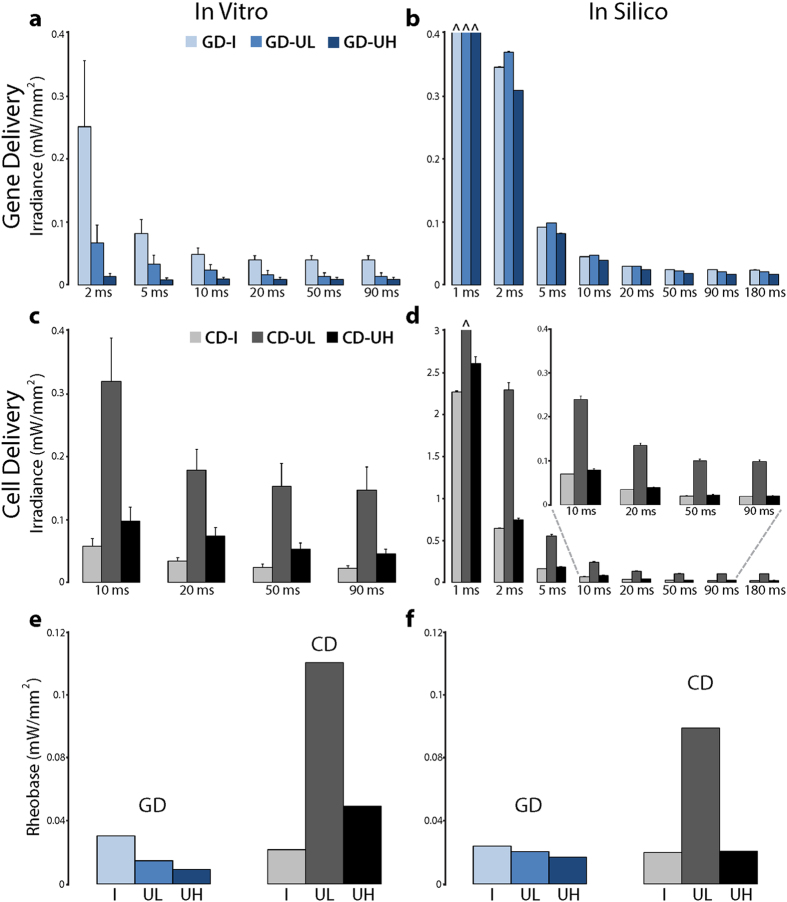
Threshold optical energy as a measure of excitability in optogenetically-modified cardiac syncytia. Strength-duration relationships for *in vitro* monolayers with light-sensitivity inscribed by (**a**) GD and (**b**) CD (n = 5–17 for pulse widths 2–90 ms). Analogous strength-duration relationships for *in silico* monolayers with light-sensitivity inscribed by (**c**) GD and (**d**) CD (n = 5 for pulse widths 1–180 ms). (**e,f**) Averaged *in vitro* and *in silico* rheobase values derived from optical strength-duration curves in (**a–f**).

For each combination of transgene delivery mode and spatial pattern, we used light pulses with controlled irradiance levels to identify the threshold for optical excitation, both *in vitro* and *in silico*. This allowed us to quantitatively characterize the relationship between transgene properties and the efficiency of gene or cell therapy (e.g., for restoration of excitability). *In vitro* strength-duration curves were constructed for pulse widths ranging from 2 to 90 ms with irradiance values of up to 0.6 mW/mm^2^ (CD-UL) (Fig. 6). For GD (Fig. 6a), optical excitation thresholds were higher for the island configuration (GD-I) than for uniform distributions (GD-UL and GD-UH). That is, a localized GD distribution did not yield any special advantages and, for uniform patterns, maximizing opsin density in GD monotonically increased optical excitability. Notably, the proportion of ChR2-expressing cells in GD-UH configurations (approximately 71%, Fig. 3c) was greater than that in the densest areas of GD-I patterns (approximately 62%, Fig. 3a), which provides a direct explanation for the fact that the latter were less optically excitable than the former. Generally speaking, GD-UL configurations had lower excitation thresholds than GD-I patterns, even though their overall proportion of light-sensitive cells was lower (approximately 36%, Fig. 3b); this suggests that distribution of ChR2-rich cells throughout the illuminated area instead of a compact central region resulted in a configuration that was less susceptible to source-sink mismatch effects during optogenetic stimulation.

For CD configurations (Fig. 6c), the island pattern (CD-I) resulted in the lowest optical excitation thresholds, but uniform distribution with high donor cell density (CD-UH) was also highly excitable. In general, for CD, optical excitability was improved by the spatial consolidation of transgene-expressing donor cells for generating maximum charge upon light stimulation. Strength-duration experiments conducted *in silico* using an expanded range of light pulse widths (Fig. 6b,d) reproduced the *in vitro* behaviour associated with each combination of delivery mode and transduced cell spatial pattern. The relative excitation threshold values were consistent with those measured *in vitro*, and, importantly, the same general relationships between E_e,thr_ and spatial patterns were observed *in silico* and *in vitro*. This similarity is best appreciated by examining *in vitro* and *in silico* rheobase values for each configuration (Fig. 6e,f), which were derived from strength-duration curves in Fig. 6a–d. Within the UL and UH configurations, the rheobase was higher for CD than GD, reflecting the generally higher excitation thresholds for CD in this system.

For CD syncytia, quantitative agreement between *in vitro* and *in silico* optical excitation thresholds was only achieved when computational models incorporated the 3D distribution of donor cells observed via Z-stack imaging (Fig. S1). The largest effect of 3D geometry on functional behavior was for CD-I cases, in which a large proportion of ChR2-expressing donor cells were located on top of (and electrically coupled to) a layer of myocytes, expanding the conductive interface between light-sensitive and excitable tissue regions. In an earlier study of cardiac tissue light-sensitized by CD of ChR2^25^, we showed that the optical energy required to excite was lower for distributions that had a large and spatially-consolidated interface between donor cells and myocytes compared to patterns with smaller, more diffuse donor cell clusters. Here, we quantitatively characterized this “consolidated interface” property by calculating a metric of spatial autocorrelation, Moran’s I, for each model. Consistent with our previous findings, CD-UL models, which were associated with the highest E_e,ethr_ values, had much lower Moran’s I values (I = 0.357 ± 0.009) compared to models that were more easily excited by optical stimulation (I = 0.809 ± 0.011 and I = 0.911 ± 0.001 for CD-UH and CD-I, respectively). This explains why E_e,thr_ values for CD-I cases were among the lowest observed in all experiments.

### General principles linking delivery mode and spatial distribution with optical excitation threshold

After establishing that results of optical stimulation in models were consistent with those observed *in vitro*, we sought to uncover general spatial determinants of functional efficiency (ability to increase/restore excitability) for GD and CD transgene delivery approaches, as quantified by the optical threshold for excitation (specifically, E_e,rheo_ in Fig. 6e). Simulations were carried out using a wider range of transgene configurations, incorporating light-sensitive cell spatial distributions with extremely low densities and clustering patterns that would have been difficult to study experimentally. This allowed for a finer scanning of the parameter space by quantifying optical excitability in 40 additional models with unique combinations of delivery mode and transgene spatial distribution (Fig. S3,S4). Transgene spatial distributions were quantitatively characterized using two metrics. The centre density metric (CDM) was defined as the proportion of opsin-expressing cells in the area within a 1.5 mm radius at the centre of the sample. The interface metric (IM) was Moran’s I^29^, a measure of spatial autocorrelation, which quantified the extent of interface between transduced and non-transduced cells in the entire sample; see **Materials and Methods** for details regarding calculation and qualitative meaning of IM.

**Figure 7:**
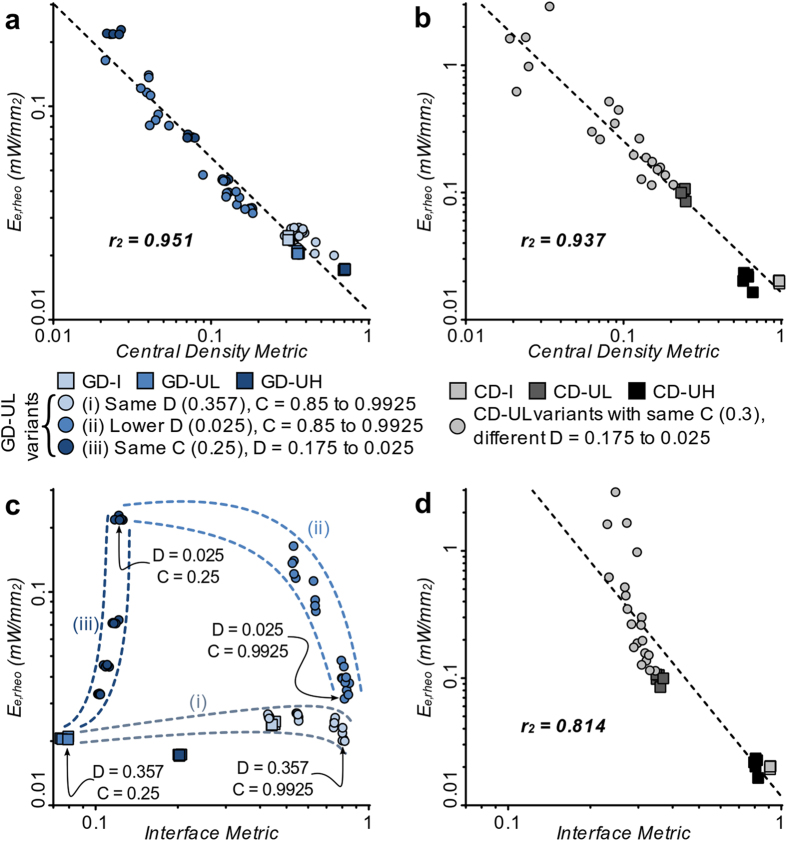
Generalized relationships between transgene patterns and optical excitability. (**a**) Threshold irradiance rheobase (E_e,rheo_) compared to central density metric (CDM) for light sensitive cell distribution in GD models. Along with transgene expression patterns considered in Figs 1, 2, 3, 4, 5, 6 (GD-I, GD-UL, and GD-UH), additional simulations were conducted in models with other combinations of D and C (Fig. S3). Simple linear regression on log-log transformed data revealed an apparent power law relationship. (**b**) Same as (**a**), but for CD models (see also: Fig. S4). (**c**) E_e,rheo_ compared to the interface metric Moran’s I (IM) for GD models. No overall trend was observed; however, dashed lines highlight general patterns in interface/excitability relationship (see text). (**d**) Same as (**c**) but for CD models. For this case, there was an apparent power law relationship as in (**a**,**b**).

Linear regression on log-log transformed data (Fig. 7a,b) revealed apparent power law relationships between E_e,rheo_ and CDM for both GD (E_e,rheo_ = 0.0110 × CDM^–0.72^; correlation coefficient r = –0.975) and CD (E_e,rheo_ = 0.0164 × CDM^–1.19^; r = –0.968). Thus, for both delivery modes, functional efficiency of transgene expression improved monotonically (E_e,rheo_ decreased) with higher transgene density. In contrast, the relationships between functional efficiency and IM differed considerably for GD and CD (Fig. 7c,d). For CD (Fig. 7d), consolidation of transgene-expressing donor cells (as indicated by higher IM values) was associated with improved functional efficiency (lower E_e,rheo_); this resulted in an apparent power law relationship (E_e,rheo_ = 0.0118 × IM^–2.64^; r = –0.902) similar to those observed for CDM above. For GD, the effect of transgene consolidation was more complex (Fig. 7c). Although no overall trend was observed, analysis revealed general sub-patterns in the E_e,rheo_ vs. IM relationship (dashed lines in Fig. 7c). When transgene expression density was high (D = 0.357), excitability was insensitive to changes in IM due to increased clustering (Fig. 7c, i). In contrast, when expression density was low (Fig. 7c, ii: D = 0.025), higher IM was associated with improved functional efficiency, with near order of magnitude difference between E_e,rheo_ values for the diffuse and clustered distributions. Finally, when the spatial distribution of light-sensitive cells was diffuse (Fig. 7c, iii: low IM due to C = 0.25), the functional efficiency of GD-transduced syncytia was exquisitely sensitive to transduction density.

In summary, this analysis revealed a straightforward relationship between higher functional efficiency (i.e., lower optical excitability) and higher transgene density for both GD and CD. For a subset of configurations (all CD cases and low-density GD-UL patterns), increased clustering of transduced cells also strongly improved optical excitability.

### Validation of the optogenetics-based strategy to quantify the efficiency of gene and cell therapy for restoration of cardiac excitability

**Figure 8:**
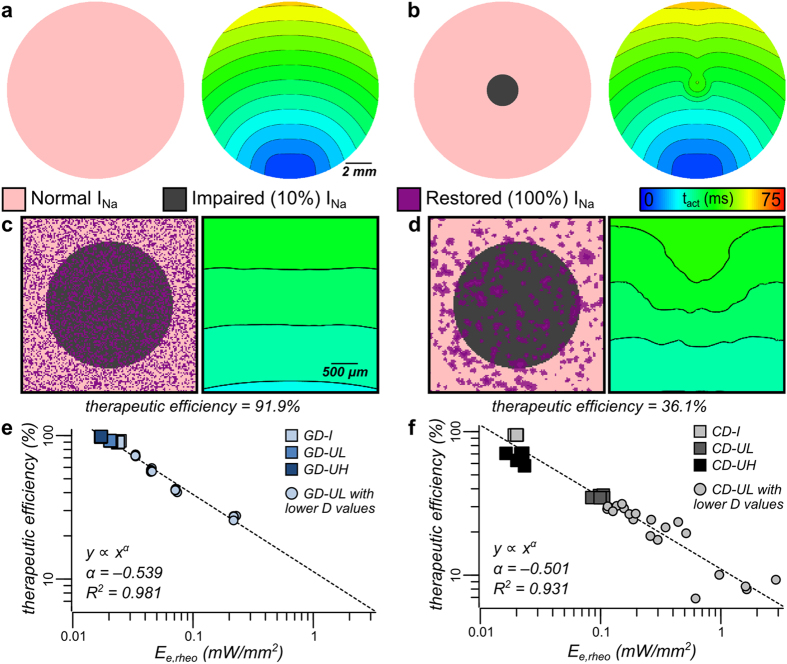
Validation of the proposed optogenetics-based strategy to quantify the efficiency of gene and cell therapy. (**a,b**) Schematics (for control and fully impaired cases, respectively) showing spatial distribution of normal/diseased tissue (left) and resulting activation patterns (right). (**c**) Zoomed-in views of the same maps as in (**a**), but for a case where excitability restoration was attempted via GD of I_Na_ to a subset of myocytes in a spatial pattern corresponding to GD-UL; therapeutic efficiency was calculated by comparing the total activation time to those for control (**a**) and fully impaired (**b**) cases, as described in the text. (**d**) Same as (**c**), but with excitability restored via delivery of I_Na_-rich myocytes in a CD-UL spatial distribution. (**e,f**) Demonstration (for GD and CD, respectively) that the therapeutic efficiency of excitability restoration via delivery of I_Na_ to a particular set of cells is related by an inversely proportional power law to the optical energy required to excite optogenetically-transduced cells with an identical spatial pattern.

We sought to demonstrate that the optical energy needed to excite light-sensitized cardiac tissue with a particular optogenetic configuration can be used to approximate the efficiency of restoring excitability via gene or cell delivery with the same spatial pattern. The developed *in silico* tools were used for this purpose. A “healthy” control model was created, with normal myocytes throughout (Fig. 8a), and an “impaired” case was also generated—with severely impaired excitability (10% of normal sodium current, I_Na_) in a central disc-shaped region (2.5 mm diameter). The size of this central region approximated the island size for GD/CD-I expression patterns (see Fig. 2a,d). Activation sequences (right-hand side panels of Fig. 8a,b) resulting from electrical stimulation (same protocol as in Fig. 4) were constructed and in each case the activation time of the entire central region (*AT*) was obtained: *AT*_*ctrl*_* *=* 10.7 ms* and *AT*_*imp*_* *=* 19.1 ms* for Fig. 8a,b, respectively. Reduced excitability caused a prominent decrease in CV, as evidenced by the local curvature of isochrones near the central region in Fig. 8b.

The “therapy” to restore excitability in the impaired model involved either restoring normal I_Na_ in a subset of cells (GD) or inserting donor cells with normal levels of I_Na_ (CD). For both delivery modes, we used spatial patterns identical to the optogenetic GD or CD configurations (i.e., distributions from Fig. 3a–f). For completeness, additional spatial distributions of transduced cells were simulated that had the same degree of clustering as GD-UL and CD-UL patterns but with progressively decreasing densities (*D *=* 17.5%, 12.5%, 7.5%, 2.5%*). Notably, donor cells for CD cases in this set of simulations were modeled as myocytes and had normal electrical coupling to host cells, as may be relevant to currently pursued therapies, e.g., injection of cardiosphere-derived cells to restore excitability to an infarcted region^9^. For each unique combination of delivery mode and spatial pattern, the associated activation time for the central region (AT_x_) was determined for electrical stimulation; then, the therapeutic efficiency of excitability restoration was quantified as *(AT*_*imp*_ *– AT*_*x*_*)/(AT*_*imp*_ *– AT*_*ctrl*_). Examples of restoration attempts with GD-UL and CD-UL patterns are shown in Fig. 8c,d; the respective central activation times for these two cases were 11.4 and 16.1 *ms*. Activation maps were qualitatively consistent with our quantitative assessment of therapeutic efficiency; for example, the CD-UL case shown here (therapeutic efficiency of only 36.1%) had distorted isochrones in the central region due to poor restoration of excitability.

We derived generalized relationships (Fig. 8e,f, corresponding to GD and CD, respectively) between therapeutic efficiency and the optical energy required to excite ChR2-expressing GD or CD models with the identical spatial distribution of transduced cells (using E_e,rheo_ values from Fig. 6e). This analysis demonstrated that, regardless of delivery mode, there was a strong inverse relationship between therapeutic efficiency and E_e,rheo_ values associated with different spatial patterns. Least-squares regression (to fit functions of the form y = Ax^α^) showed that both relationships follow power law curves, with similar Statement revised to be consistent with corrected data in Fig. 8e. scaling exponents (α values) indicated a steeper regression slope for CD than for GD; in both cases, Pearson’s correlation coefficient was high (R^2^ = 0.981 and 0.931 for GD and CD, respectively), which suggested that E_e,rheo_ had clear predictive value. For all CD-UL cases examined here, the resulting restoration of excitability was rather poor (therapeutic efficiency <40%), consistent with the fact that these types of configurations were associated with the highest optical excitation thresholds observed *in vitro* or *in silico*.

## Discussion

In this study, we developed a new methodology for testing structure-function relationships in the context of gene and cell therapy in cardiac tissue, focusing on the restoration of excitability. This approach uses optogenetics as a means for selective cell-specific interrogation by light, with the optical excitation threshold serving as a surrogate for therapy efficiency. Our findings are directly applicable to devising new approaches for optogenetic manipulation of cardiac tissue, as well as for optimizing the efficiency of such optical actuation methods by selecting transduction strategies (and resultant spatial patterns) that work with the lowest optical energies. However, based on presented proof-of-concept simulations, we also argue that our approach can serve as a generalized technique for testing the efficiency of gene/cell therapy for other purposes by abstracting the contribution of a range of excitatory ion channels that might be used in gene/cell therapy.

Different experimentally relevant conditions can be tested by the proposed method, including variability of dose, delivery mode, and spatial transduction pattern for the transgene of interest. Experimentally, we used transgene patterning of excitable cells (CMs) and non-excitable donor cells to design three relevant transgene distributions: (1) localized islands; (2) spatially-diffuse, low-density distributions; and (3) spatially-diffuse, high-density distributions. We describe a new general-purpose method to pattern gene and cell expression *in vitro* using viral infection in suspension, cell titration, and stencil-based shapes^18^. This is a simpler, more robust alternative to a previously reported methodology for patterning gene expression based on magnetic nanoparticles^30^. Importantly, as part of the biophysical analysis tools developed in this study, we performed side-by-side comparisons of the functional outcomes from experiments and computations using identical configurations.

While such testing could be technically done using direct transduction with a particular gene of interest, e.g., the skeletal muscle Na^+^ channel^31^ or the hyperpolarization-activated, cyclic nucleotide-gated pacemaker channel^32^, the abstracted optogenetics-based methodology used here offers unique advantages: 1) it allows for selective probing of only the transduced cells in order to quantify their functional contribution unambiguously; and 2) it allows for the derivation of general relationships about the effects of structure (spatial transgene arrangements) on functionality. An important finding of this study is that optogenetic excitability of a particular gene or cell delivery configuration has predictive value for the therapeutic efficiency of that same configuration in restoring excitability via the expression of an excitatory ion channel transgene.

This study also presented the development and validation of a novel computational framework to study optogenetic gene and cell delivery in cardiac tissue. The experimental and model results (Figs 4 and 5) are not identical, which is to be expected since there are physiological differences between the computational model and the referent system (e.g., species, exact electrophysiological properties of the cardiomyocytes and the donor cells, ChR2 expression rate, tissue-scale electrical coupling, precision with which optical stimulus strength can be adjusted). However, our models correctly reproduce experimental observations in two important ways: 1) macroscopic conduction patterns are consistent, for both electrically- and optically-elicited excitation waves; and, 2) optical excitation thresholds associated with different delivery modes and transduced cell spatial patterns are an excellent match, especially as relative threshold levels for different configurations. Therefore, the *in silico* framework is appropriate for simulation of gene/cell therapy involving optogenetics-related targeting, e.g. ChR2, and other transgenes. This approach enables: 1) the automatic generation of experimentally-relevant transgene spatial distributions and the assessment of therapeutic efficiency using optogenetics; 2) the ability to explore structure-function relationships in a rigorous way by simulating a much wider spectrum of conditions than feasible to pursue experimentally, including extension to organ-scale analysis of virtual GD and CD manipulations (Figs S3–4)^23^,^25^; and, 3) the side-by-side exploration of structure-function relationships for different transgenes (e.g., optogenetic stimulation vs. restoration of excitability, as in this study) with identical delivery modes and transduced cell spatial patterns (Fig. 8). No prior systematic attempts have been made to analyse and quantify the spatial effects of transgene delivery on the performance of the transgene. The computational tools used here provide a platform that will help answer other questions related to both optogenetic applications and to gene and cell therapy in general.

Our empirically validated computational framework also allows detailed analysis of general principles linking spatial characteristics of ChR2-expressing cell distribution patterns with the optical energy needed to elicit a propagating action potential response (Fig. 7). When generalized to gene and cell therapy for restoration of excitability, these findings are relevant to several currently employed experimental modalities of GD and CD. For example, our results indicate that cell therapy will be most effective if intramyocardial injection can be optimized to reliably generate localized, aggregated clusters of transgene-expressing cells as opposed to the low density, spatially-distributed expression pattern by systemic delivery of the same number of cells. In contrast, gene delivery is relatively insensitive to spatial clustering and delivery method (i.e., direct injection or systemic) should be chosen to maximize density/efficiency of expression. For Fig. 1-7, our findings regarding cell delivery quantitatively describe the behaviour of transgene-expressing donor cells with distinct cell- and tissue-scale electrophysiological properties from host cardiomyocytes (i.e., reduced intrinsic excitability and capacity for intercellular coupling). We expect that different donor cell types would exhibit distinct structure-function relationships. In particular, for donor cells with excitability and coupling characteristics more similar to host cells (e.g., exogenous cardiomyocytes, stem cell-derived cardiomyocytes, cardiospheres, etc.)^33^,^34^,^35^, we would anticipate a spectrum of intermediate behaviours between those observed here for the discussed limit cases of CD and GD configurations. For example, if simulations exploring inscription of light sensitivity via CD (Fig. 7b,d) were repeated using donor cells with greater excitability than HEK cells and/or the capability to form stronger electrical connections with host myocytes, we would expect to observe functional behaviour closer to that for GD configurations (Fig. 7a,c). In the present study, it is not possible to tease apart the specific contributions of cell- and tissue-scale differences in donor cell properties; future work using similar experimental tools to those developed here will be necessary to address this question.

It is important to note that our criterion for optimal transgene expression (threshold for optical excitation) was intended to represent the optimal engagement of a transgene of interest in the excitation of cardiac tissue; we did not explore potential arrhythmic effects as related to transgene distribution. Any gene or cell therapy introduces a certain degree of heterogeneity (Figs 2 and 3) in the native tissue, which in itself can be pro-arrhythmic. Notably, in cases where transgene delivery is targeted to restore excitability in a specific compromised area, such as the peri-infarct zone in patients with acute myocardial infarction, the heterogeneous distribution of transduced cells resulting from gene/cell delivery in tissue as a whole could in fact *homogenize* electrophysiological properties, resulting in reduced inducibility of arrhythmia. Nevertheless, the results of our analysis must be interpreted carefully—a particularly clustered/aggregated transgene pattern produced by GD or CD might be very “effective” in terms of its contribution to tissue-level excitation, but it might also create or exacerbate the substrate for reentry initiation by perturbing conduction patterns, as evidenced by the irregular conduction seen for CD-I in this study (Figs 4 and 5).

For the sake of simplicity, the present study focused only on spatial aspects of gene and cell delivery. We used delivery of an excitatory opsin to mimic the contributions of any excitatory ion channel or pump (introduced by GD or CD) and we used computer simulations to confirm that this abstraction is valid. Nonetheless, many factors in addition to spatial aspects will influence transgene functionality in cardiac tissue, such as action kinetics of the expressed proteins and coupling potential of the donor cells. Furthermore, the use of a 2D *in vitro* experimental platform, admittedly a simplification of the heart’s complex environment, allowed direct quantitative comparisons and validation of the computational model. Having completed this important validation process, our modelling framework can be extended to 3D, allowing for more realistic predictions of performance, as we have shown in preliminary simulation work^25^. Experimentally, *in vivo* gene and cell therapies face many challenges; nonetheless, we believe that the general principles derived here relating to spatial arrangements and excitability will be applicable as guiding principles for *in vivo* expression of specific transgenes to restore function in the beating heart.

## Materials and Methods

### Cardiomyocyte Isolation

The experimental protocol was approved by the Institutional Animal Care and Use Committee at Stony Brook University; all experiments were performed in accordance with relevant guidelines and regulations published by this committee. Neonatal rat ventricular cardiomyocytes (NRVM) were isolated using a previously published technique^14^. In short, the ventricular apex was excised from 2–3 day old Sprague-Dawley rats and enzymatically digested overnight with trypsin (1 mg/mL; USB, Cleveland, OH), followed by collagenase (1 mg/mL; Worthington Biochemical Corporation, Lakewood, NJ) 12–14 hours later. Fibroblasts were removed by a two-stage pre-plating procedure. NRVMs were suspended in M199 media (Invitrogen, Grand Island, NY) supplemented with 12 μM L-glutamine (Invitrogen), 0.05 μg/mL penicillin-streptomycin (Mediatech Cellgro, Kansas City, MO), 0.2 μg/mL vitamin B12 (Sigma-Aldrich, St. Louis, MO), 10 mM HEPES (Invitrogen), and 3.5 mg/mL D-(+)-glucose (Sigma-Aldrich) supplemented with 10% fetal bovine serum (FBS; Invitrogen) prior to the introduction of light-sensitive channels and plating.

### Design of Ad-hChR2(H134R)-eYFP

The adenovirus containing the transgene for hChR2(H134R)-eYFP was prepared at the Stony Brook University Stem Cell Facility and based on the expression cassette of plasmid number 20940 from Addgene (Cambridge, MA)^36^. Adenoviral shuttle vectors containing hChR2(H134R)-eYFP were constructed using a ubiquitous CMV promoter on a pBR322 backbone^37^ and subsequently used to construct recombinant first generation adenovirus by homologous recombination into pTG3602^38^. Virus genomes were transfected into and propagated in HEK293 cells and purified by CsCl banding.

### Preparation of ChR2-Expressing NRVMs and Donor Cells

Adenoviral infection of NRVMs was completed as previously described^18^. In short, after isolation NRVMs were infected with an optimized dose of virus (multiplicity of infection, MOI 25). Cells were incubated during infection at 37 °C for two hours in 2% FBS-containing M199 media with manual agitation every 15–20 minutes. After infection cells were resuspended in 10% FBS-containing M199 media. Over 95% efficiency of cardiomyocytes expressed ChR2 as confirmed by eYFP visualization and quantification. For experimental cell delivery simulations, a ChR2-expressing HEK293 cell line was developed as previously described^14^ using plasmid number 20940 from Addgene (hChR2(H134R) –eYFP)^36^. As reported previously, presence of the transgene was confirmed in passages 2 through 20 by both reporter protein expression and functional testing^14^.

### Design of Spatially Distinct Transgene Distributions

In order to mimic physiologically relevant transgene distributions achievable with both cell and viral gene delivery approaches, we experimentally created three spatially-distinct transgene distributions: a spatially-localized island, a spatially-uniform low density monolayer, and a spatially-uniform high density monolayer (Fig. 2a–f). Islands (I) of ChR2-expressing cells were created using custom-made silicone elastomer stencils, where light-sensitive cells were plated in a 3 mm diameter circular area and normal cells were plated surrounding the central island. Uniform distributions of varying densities (low, UL; high, UH) were created by mixing light-sensitive and unmodified cells with fixed ratios (i.e. 35% light-sensitive and 65% unmodified for GD-UL)^18^.

### Optical Mapping of Light-Sensitive Syncytium

Cell monolayers were stained with the calcium-sensitive dye Quest Rhod4 AM (10 μM; AAT Bioquest, Sunnyvale, CA) diluted in Tyrode’s solution containing the following in mM: NaCl, 135; MgCl_2_ 1; KCl, 5.4; CaCl_2_, 1.5; NaH_2_PO_4_, 0.33; glucose, 5; and HEPES 5 adjusted to pH 7.4 with NaOH. All experiments were conducted at 30 ± 0.5 °C. The macroscopic imaging system used for fluorescent imaging of NRVM monolayers has been described previously^14^. The system includes a CMOS camera (200 frames/sec over 1,280 × 1,024 pixels; pco, Germany); a Gen III fast-response intensifier (VideoScope International, Dulles, VA); light collection optics (Navitar Platinum lens, 50 mm, f/1.0) and emission filter (610/75 nm); an excitation light source (525 nm; Oriel, Stratford, CT); and an adjustable imaging stage. Propagation data was collected using the commercially-available software CamWare (pco, Germany). Data was spatially and temporally filtered, using the Bartlett and Savistsky-Golay filters, respectively, before being analyzed in custom-developed Matlab (Mathworks, Natick, MA) software.

During optical mapping, cardiac monolayers were electrically stimulated using custom-built bipolar platinum electrodes driven by a Myopacer Cell Stimulator (IonOptix, Milton, MA). Optical pulses for light-triggered stimulation were delivered globally (i.e. via illumination of the entire monolayer) using a fiberoptics-coupled diode-pumped solid-state (DPSS) laser (470 nm; Shanghai Laser, Shanghai, China). Energies for optical stimulation were reported in terms of irradiance (mW/mm^2^) and measured using a digital optical power meter (Newport, Irvine, CA).

### ChR2(H134R) Localization and Quantification

Monolayers were fixed with 3.7% formaldehyde, permeabilized with 0.2% Triton-X 100, and stained with the monoclonal mouse antibody for sarcomeric α-actinin (Sigma-Aldrich, St. Louis, MO) and the goat anti-mouse secondary antibody conjugated to Alexa-Fluor 647 (Invitrogen). Panoramic confocal imaging was performed to quantify transgene expression (based on the eYFP reporter fused to ChR2) using an Olympus Fluoview FV1000 system. Thresholds for transgene expression were determined using threshold binarization methods^39^ to create templates for *in silico* analysis.

### Computational Models of Light-Sensitive Syncytia

All simulations were conducted in models based on a 14 mm-diameter 25 μm-height 3D disc geometry, chosen to match *in vitro* conditions. A higher mesh resolution (hexahedral element edge length: 25 μm; Table S1) was used compared to typical cardiac electrophysiology models^40^,^41^ to match fine-scale features observed experimentally. Myocyte membrane kinetics were represented by the Luo-Rudy (LR1) action potential (AP) model^42^; alternative AP models based on measurements from neonatal rat ventricular myocytes do exist (e.g., Korhonen *et al.*^43^) but the LR1 formulation is much more computationally efficient and captures the same essential excitability features (i.e., depolarization driven by fast sodium current). Using the LR1 model allowed us to explore a large parameter space (via the execution of 6704 unique simulations) in an acceptable time frame (Table S1). Donor cell membrane kinetics were simulated using an electrically passive model as described previously^25^. Model parameters in donor tissue for resting membrane voltage, specific membrane conductance, and cell radius were chosen as –50 mV, 14 μS/cm^2^, and 6.5 μm, respectively. These values, which are within the physiological range for generic small inexcitable cells, e.g. HEK cells^14^,^44^, were chosen to match experimental *in vitro* results for CD cases.

Excitation propagation was governed by the monodomain formulation, with isotropic conductivity defined by the harmonic mean of intracellular and extracellular conductivities (σ_i,x_ for tissue type *x* and σ_e_ = 0.625 S/m, respectively)^45^ using the CARP software package (Johns Hopkins University, Université de Bordeaux, Medizinische Universität Graz)^46^,^47^. Contiguous myocardial regions were modeled with σ_i,myo_ = 20.25 mS/m to approximately match conduction velocities (24.3 ± 0.7 cm/sec) measured in neonatal rat ventricular myocyte monolayers^48^; for clusters of donor cells, this value was reduced by 80% (σ_i,donor_ = 0.2 × σ_i,myo_ = 4.05 mS/m), to approximate *in vitro* behavior of donor cells^14^,^25^. To represent reduced heterocellular (donor-myocyte) coupling, we first electrically isolated all donor cell regions using the discontinuous finite element approach described by Costa *et al.*^49^; then, current flow across each cut face was governed by a resistive connection (G_hetero_ = 0.25 μS) at 10% of the nominal cell-cell electrical coupling in myocardium^50^.

### Simulation of Light-Sensitive Current in Computational Models

Inscription of light sensitivity in either myocytes or donor cells was simulated by adding the most recent published model of light-sensitive I_ChR2_^16^ to the total cellular transmembrane current, as outlined in by Boyle *et al*.^25^. All I_ChR2_ model parameters were as published; maximal channel conductance (g_ChR2_) was increased two-fold for CD and six-fold for GD (0.4 mS/μm^2^ to 0.8 mS/μm^2^ and 2.4 mS/μm^2^, respectively) to achieve a better match between simulated and experimentally-measured E_e,thr_ values. These non-matching optimal g_ChR2_ values identified for GD and CD reflected the variability of ChR2 expression efficiency between cardiomyocytes and inexcitable donor cells.

### Simulated Distribution of Light-Sensitive Cells in Computational Models

Here, we present a condensed summary of the methodology used to achieve a good match between light sensitive cell distributions observed *in vitro* and those simulated *in silico*. A comprehensive explanation of this process, including a list of the parameters used to generate each spatial pattern and a description of all regularization steps, can be found in the Supplementary Materials and Methods section.

Using the methodology described by Boyle *et al*.^25^, ChR2-expressing cells were distributed in monolayer models by a stochastic algorithm with parameters controlling density by volume (D) and clustering (C) of light-sensitive cells/elements. Increasing C resulted in a progression from highly diffuse (C≈0) to highly clustered (C≈1) spatial patterns. To match each class of ChR2 patterns created *in vitro*, D was chosen based on the empirical proportion of light-sensitive cells; then, C was iteratively adjusted to obtain qualitative (compare Fig. 2a–f with Fig. 3a–f) and quantitative (Fig. 3g,h) agreement between imaged and generated distributions. Additional regularization steps (identification of monolayer sub-regions, limiting of cluster size, erosion/dilation filtering) were applied to achieve close correspondence between generated models and *in vitro* patterns. In total, 30 models were generated to match *in vitro* distributions (2 delivery modes, 3 patterns, n = 5). Additional models (n = 80) with a wider variety of light-sensitive cell distributions enabled a more comprehensive analysis of excitability properties for the two delivery modes (Figs S3–4), not feasible or extremely demanding to perform *in vitro*.

### Protocol for Conducting Simulations in Computational Models

For each unique light-sensitive model described above and a control model without ChR2 delivery, ten pre-conditioning electrical stimuli (strength: 72 μA/cm^2^, duration: 2 ms, basic cycle length (BCL): 1000 ms) were applied to a 2.4 mm by 1.2 mm rectangular area (white dashed line in Fig. 4b) to achieve steady state. After the 11^th^ electrical stimulus, conduction velocity (CV) was determined by differencing local activation times along the direction of wavefront propagation at points ± 3 mm from the center of the disk, minimizing boundary effects. Optical stimulation was applied uniformly, as done *in vitro*. For each of the pulse durations used *in vitro* (t_stim_ = 1, 2, 5, 10, 20, 50, 90 ms) and an additional longer one for rheobase estimation (t_stim_ = 180 ms), the threshold irradiance for a propagating response (E_e,thr_, mW/cm^2^) was identified using a bisection approach. For *in silico* activation maps in Figs 4 and 5, activation times were determined by identifying the instant of threshold crossing in the membrane voltage signal (threshold V_m_ = –20 mV); to match the methodology used *in vitro*, activation maps were interpolated using a moving average filter with a circular kernel (125 μm radius).

### Quantitative Characterization of Light-Sensitive Cell Spatial Patterns in Computational Models

To provide a quantitative estimate of the extent of interface between light-sensitive cell clusters and regions of normal myocardium, we chose to use Moran’s I, a simple measurement of spatial autocorrelation^29^, given by Equation (1):


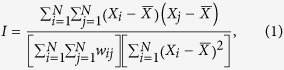


where N is the total number of elements in the sample, X_ζ_ is the status of the ζ^th^ element (1 for ChR2-expressing, 0 for normal myocyte), X is the mean value of X_ζ_ for all N elements, and w_ij_ are entries in a connectedness matrix (w[i, j] = 1 if elements i and j are neighbors, otherwise 0). General metric behavior is as follows:

- I = –1 for a perfectly structured diffuse (non-clustered) pattern, i.e. an alternating “checkerboard” distribution of ChR2-expressing and non-expressing cells;
- I ≈ 0 for a random diffuse pattern; and,
- I = 1 for a perfectly clustered/aggregated pattern, i.e. a contiguous region of ChR2- expressing cells separated from non-transduced cells.

Execution of the distribution algorithm with high C values yielded distributions with a high value of Moran’s I and vice-versa.

### Statistical Analysis

For both *in vitro* and *in silico* results, all quantitative data are presented as mean ± standard error of the mean. Statistically significant differences were identified using ANOVA followed by Tukey-Kramer’s test with a significance level of p < 0.05.

## Additional Information

**How to cite this article**: Ambrosi, C. M. *et al.* Optogenetics-enabled assessment of viral gene and cell therapy for restoration of cardiac excitability. *Sci. Rep.* **5**, 17350; doi: 10.1038/srep17350 (2015).

## Supplementary Material

Supplementary Information

Supplementary Movie S1
