# Supplementary material for: Optogenetics-enabled assessment of viral gene and cell therapy for restoration of cardiac excitability
Source: Sci Rep. 2015 Dec 1;5:17350. doi: 10.1038/srep17350 (PMC4664892; doi:10.1038/srep17350)
Supplement: Supplementary Information [file srep17350-s1.pdf]

Optogenetics-enabled assessment of viral gene and cell therapy for restoration of cardiac excitability

Christina M. Ambrosi, PhD[1*], Patrick M. Boyle, PhD[2*],
Kay Chen, BS,[1] Natalia A. Trayanova, PhD,[2] Emilia Entcheva, PhD[1**]

1: Department of Biomedical Engineering, Stony Brook University, Stony Brook, NY
2: Institute for Computational Medicine, Johns Hopkins University, Baltimore, MD

*: The first two authors contributed equally to this work

**: Corresponding Author: Emilia Entcheva, PhD, Phone 631-444-2368,
Email: emilia.entcheva@stonybrook.edu, Mail: Institute for Molecular Cardiology,
Stony Brook University BST-6, Room 120B, Stony Brook NY 11794-8661, USA

SUPPORTING INFORMATION

Supplementary Materials and Methods

Stochastic Algorithm for Distribution of Light-Sensitive Cells in Computational Models

Following the procedures outlined in Boyle, et al.,[1] spatial distributions of ChR2-expressing cells in light-sensitive monolayers were simulated using a stochastic algorithm originally designed to model fibrosis.[2] Algorithm inputs were the density (D) and clustering (C) parameter values of the light-sensitive cells/elements. In a sequential manner, elements were tagged as light-sensitive one by one until the proportion of ChR2-expressing tissue (by volume) within the target region was ≥ D; 1–C was the probability that a newly-tagged element would start a new cluster. Notably, we chose to replace the "patchiness" (P) parameter from the original papers[1,2] with C = 1 – P to ensure an intuitive relationship between input values and generated patterns. With this change, higher values of C (≈1) resulted in clustered spatial patterns of ChR2-expressing cells; lower values of C (≈0) resulted in diffuse distributions.

Tailoring Computational Models to Better Match Experimental Data

For each class of ChR2 distributions created *in vitro* (I, UL, and UH) and for both delivery modes (GD and CD), the density (D) parameter value was chosen by directly measuring the proportion of light-sensitive cells in imaged monolayers; then, C was iteratively adjusted to ensure that models generated by the distribution algorithm and imaged monolayers had similar patterns, as determined by comparison of normalized frequency histograms (**Figures 3g-h** for *in vitro* and *in silico*, respectively). For the experimentally constrained optimal D and C parameters, numerous *in silico* samples (n = 5) were generated by the stochastic algorithm for each of the six cases examined *in vitro*. Several additional regularization steps were applied to ensure close correspondence between *in silico* and *in vitro* models:

- **GD cases:** For GD-UL and GD-UH, the distribution algorithm was applied as described previously [1] to the entire monolayer with parameters determined as described above (GD-UL: D = 0.357 and C = 0.25; GD-UH: D = 0.709 and C = 0.8). For GD-I, gene patterning *in vitro* exhibited complex graded distributions; realistic models were generated as follows: A primary

central region (blue-shaded in **Figure 3a**) was defined by applying the stochastic algorithm to the whole monolayer with D = 0.01306 and C = 1. A secondary "ring"-shaped region (red-shaded in **Figure 3a**) was generated by re-running the algorithm with D = 0.02939 and C = 1, then subtracting the already-generated central region. Finally, the distribution algorithm was applied another three times, with $D_A = 0.618$ & $C_A = 0.85$ in the primary central region (blue), with $D_B = 0.367$ & $C_B = 0.5$ in the ring region (red), and with $D_{outer} = 0.0125$ & $C_{outer} = 0.5$ in the remaining tissue; the total effective density was D = 0.0262.

- **CD cases:** Individual ChR2-expressing donor cell clusters were limited to a maximum area of 0.05 mm^2 and the algorithm was modified to favor the generation of ellipsoid clusters; this was to account for the fact that large, non-ellipsoid clusters were seldom observed *in vitro*. The latter regularization was achieved as follows: when an element in a particular cluster was tagged as light sensitive, each element along the *perimeter* of that cluster also had a 50% chance of being tagged. To further match the higher aggregation seen in CD samples *in vitro* (i.e., the lack of speckling), successive post-processing erosion (removal of protruding elements) and dilation (recovery of borders) steps were applied after the generation of each distribution. As a result, 97% of clusters with area less than 3125 μm^2 were removed. For generation of the CD-UL and CD-UH samples, the CD-version of the distribution algorithm, including post-processing (spatial filtering), was applied as described above (CD-UL: D = 0.225 & C = 0.3, CD-UH: D = 0.588 & C = 0.72). For generation of the CD-I samples, a central consolidated region of ChR2-expressing cells (blue-shaded in **Figure 3d**) was defined by applying the CD distribution algorithm (without cluster size regularization) to the whole monolayer with $D_{inner} = 0.045$ & $C_{inner} = 1$. Subsequently, the CD distribution algorithm was applied with $C_{outer} = 0.5$ to increase overall density to D = 0.0703, then the above-described spatial filters were applied.

A total of 30 models were generated based on actual distributions created *in vitro* (2 delivery modes, 3 classes, n = 5). Additionally, *in silico*, we generated 40 new models by varying parameters as follows: maintaining the same P-values as GD-UL and CD-UL, density D was varied (2 delivery modes, 4 D values

– 0.175, 0.125, 0.075, 0.025, n = 5), with the two lowest D values corresponding to the overall D values for CD-I and GD-I, respectively. These additional models were used to help generalize relationships between spatial distribution of ChR2-expressing cells and optical excitability properties for the two delivery modes.

Three-Dimensional "Mushroom Cap" Effects In Computational Models

In GD cases, models consisted of a single layer of elements as described above. For CD samples *in vitro*, based on three-dimensional confocal imaging, we detected unique architecture, with donor cells often forming a new layer extending from a bottom cluster ("stem") to a larger "cap" region (**Figure S1** and **Movie S1**) – a "mushroom"-shaped structure due to growth/division of donor cells during culture. Computationally, this 3D architecture was captured by vertically extending the algorithm-generated clusters and matching the difference in *width* for "stems" compared to "caps" from images obtained *in vitro* (≈65%). In CD-I models, electrical coupling between donor cells and myocytes was modeled radially (around stems) and vertically (between caps and myocytes underneath); in CD-UL and CD-UH models, only radial coupling was modeled, ignoring coupling of myocytes with the much smaller caps in these cases. Including these features helped match the functional responses of the samples.

References and Notes

1. Boyle, P. M., Williams, J. C., Ambrosi, C. M., Entcheva, E. & Trayanova, N. A. A comprehensive multiscale framework for simulating optogenetics in the heart. *Nat Commun* **4**, 2370, (2013).

2. Comtois, P. & Nattel, S. Interactions between cardiac fibrosis spatial pattern and ionic remodeling on electrical wave propagation. *Conf Proc IEEE Eng Med Biol Soc* **2011**, 4669-4672, (2011).

3. Bishop, M. J. & Plank, G. Representing cardiac bidomain bath-loading effects by an augmented monodomain approach: application to complex ventricular models. *IEEE Trans Biomed Eng* **58**, 1066-1075, (2011).

Supplementary Figures and Tables

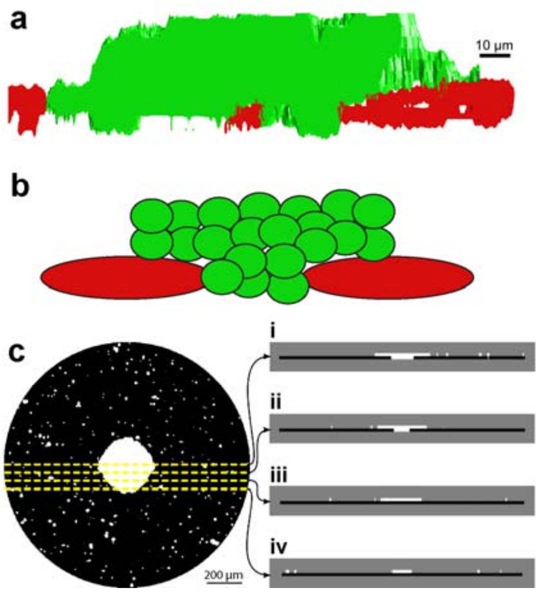

Figure S1. Three-dimensional effects of donor (HEK) cells in cell delivery. (**a**) Representative confocal z-stack of a cluster of donor cells (green) and cardiomyocytes (red). In all cell delivery configurations, confocal imaging revealed that donor cells formed additional cell layers extending in the z-dimension, as well as overlapping with surrounding cardiomyocytes. (**b**) Schematic representation of the three dimensionality of the donor cells forming a "mushroom"-shaped structure. Although ChR2-expressing donor cells and cardiomyocytes were intentionally plated as a monolayer, the proliferative nature of HEK cells resulted in growth/division during culture and prior to functional experiments and fixation for structural analyses. (**c**) Computational representation of the three dimensionality of CD in the island configuration. The unique 3D architecture was captured by modeling a "stem" and a "cap" to account for the HEK cell proliferation. The panels (i-iv) at right show cross sections at locations 50 µm apart throughout the central island of HEK cells. Panels i and ii include both "stem" and "cap", whereas panels iii and iv include only the "cap" captured towards the edge of the cluster. Differences in diameters between the stem and cap were measured to be 3.1-3.3x. The incorporation of these three dimensional features of the donor cells in the cell delivery configuration allowed for a better correlation between *in vitro* (**a, b**) and *in silico* models (**c**).

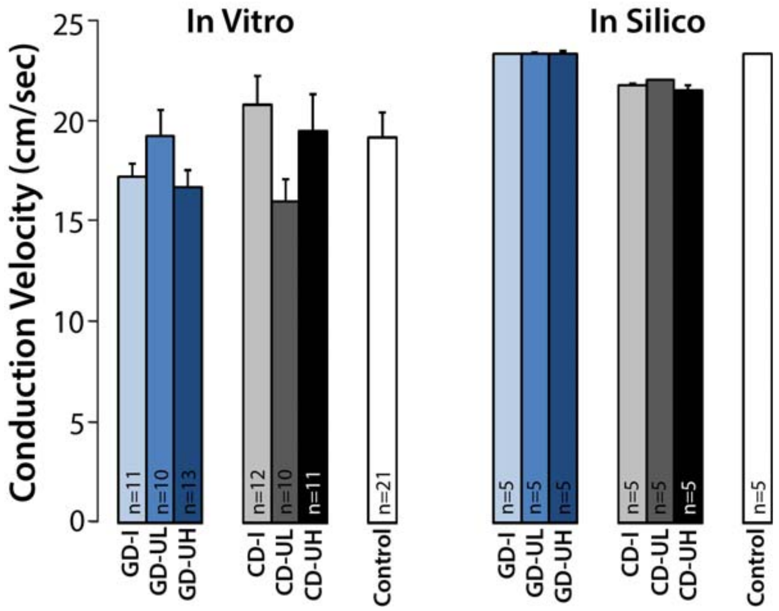

Figure S2. Conduction velocities (CVs) from the six distinct spatial distributions. Data are presented as mean ± standard error of the mean. *In vitro* CVs were collectively 18.4±0.7 cm/sec across all GD and CD configurations (at 30±0.5°C) and *in silico* CVs were 22.7±0.3 cm/sec. There were no statistically significant differences detected across the six configurations and delivery modes.

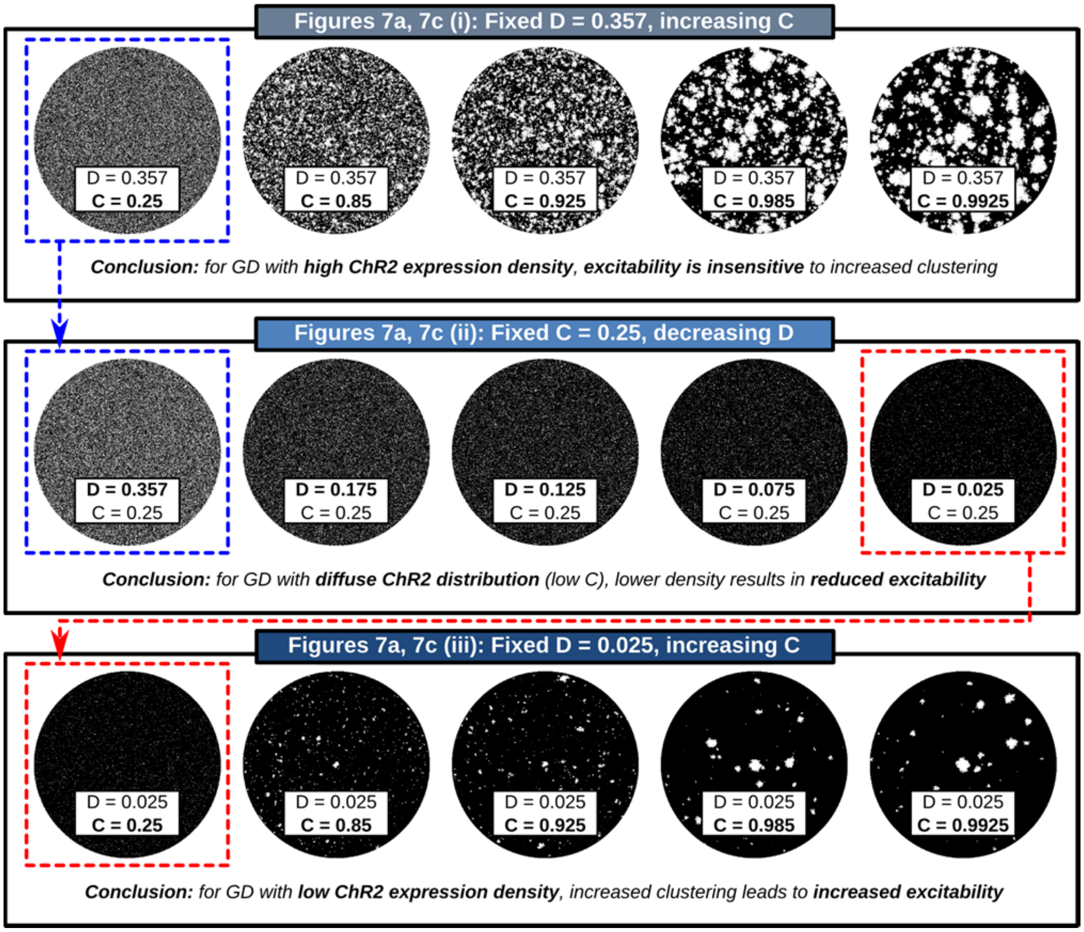

Figure S3. Additional in silico gene delivery (GD) models. Simulations were conducted on an extended set of *in silico* gene delivery models that were not experimentally feasible. The inclusion of these additional configurations allowed for the more detailed exploration of the relationship between spatial properties such as density (D) and clustering (C) of ChR2-expressing cells and optical excitability, as shown in **Figure 7a and Figure7c**. Excitability (represented by $E_{e,rheo}$) was quantified (n=5 for each additional configuration) as related to the Central Density Metric and Moran's I. Top panel (i) shows additional samples with a fixed high D (0.357) with increasing C (0.25 to 0.9925). Middle panel (ii) introduces samples with a fixed low C with decreasing D (0.357 to 0.025). Bottom panel (iii) shows samples with a fixed low D (0.025) with increasing C (0.25 to 0.9925).

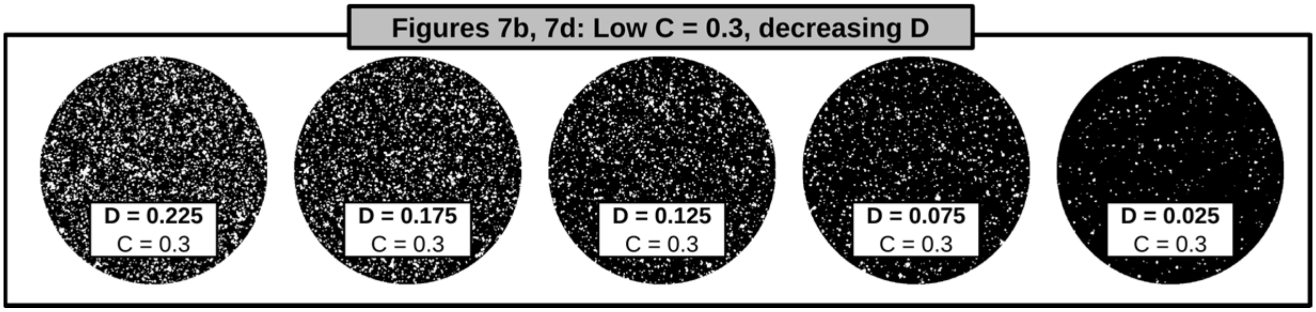

Figure S4. Additional in silico cell delivery (CD) models. Simulations were conducted on an extended set of *in silico* cell delivery models that were not experimentally feasible. The inclusion of these additional configurations allowed for the more detailed exploration of the relationship between spatial properties such as density (D) and clustering (C) of ChR2-expressing cells and optical excitability as shown in **Figure 7b and Figure7d**. Excitability (represented by $E_{e,rheo}$) was quantified (n=5 for each additional configuration) as related to the Central Density Metric and Moran's I. Additional cell delivery configurations introduced samples with fixed low C and variable D (0.225 to 0.025) to the analysis.

Table S1. In silico model parameters.

Category	Parameter(s)	Value
Geometry of disc-shaped monolayer	Number of nodes	492,482
	Number of hexahedral elements	245,124
	Element edge length	25 *μm*
Isotropic monodomain electrical conductivity *As in Bishop et al.*[3]	Myocardial tissue regions (σ_m)	19.614 *mS/m*
	Donor cell tissue regions ($\sigma_{m,donor}$)	4.0239 *mS/m*
Details regarding finite element method computation times	Number of CPUs (2.80 *GHz*)	12
	Wall time to simulate 1 second	34*m* 30*s*
	Total wall time for all simulations (n = 12,576 in 80 unique models)	72*d* 06*h* 01*m* 10*s*

Movie S1. In silico modeling of 3D features of cell delivery configurations. As described in **Figure S1**, confocal imaging revealed that donor cells formed an additional layer of ChR2-expressing cells in the z-dimension. Consequently, in order to validate *in vitro* experiments, *in silico* models needed to incorporate this unique architecture, which was captured, using a “mushroom”-shaped structure with a cap and stem. **Movie S1** shows how the model (represented in this example by CD-I) achieved this structure.
